# Curcumin and *Boswellia serrata* Modulate the Glyco-Oxidative Status and Lipo-Oxidation in Master Athletes

**DOI:** 10.3390/nu8110745

**Published:** 2016-11-21

**Authors:** Nino Cristiano Chilelli, Eugenio Ragazzi, Romina Valentini, Chiara Cosma, Stefania Ferraresso, Annunziata Lapolla, Giovanni Sartore

**Affiliations:** 1 Department of Medicine-DIMED, University of Padova, Diabetology and Dietetics, ULSS 16, via dei Colli, 4, 35100 Padova, Italy; romina.valentini@unipd.it (R.V.); stefania.ferraresso@alice.it (S.F.); annunziata.lapolla@unipd.it (A.L.); g.sartore@unipd.it (G.S.); 2 Department of Pharmaceutical and Pharmacological Sciences, University of Padova, 35100 Padova, Italy; eugenio.ragazzi@unipd.it; 3 Department of Laboratory Medicine, University of Padova, 35100 Padova, Italy; chiara.cosma@sanita.padova.it

**Keywords:** advanced glycation end-products, curcumin, inflammation, exercise, oxidative stress

## Abstract

Background: Chronic intensive exercise is associated with a greater induction of oxidative stress and with an excess of endogenous advanced glycation end-products (AGEs). Curcumin can reduce the accumulation of AGEs in vitro and in animal models. We examined whether supplementation with curcumin and *Boswellia serrata* (BSE) gum resin for 3 months could affect plasma levels of markers of oxidative stress, inflammation, and glycation in healthy master cyclists. Methods. Forty-seven healthy male athletes were randomly assigned to Group 1, consisting of 22 subjects given a Mediterranean diet (MD) alone (MD group), and Group 2 consisted of 25 subjects given a MD plus curcumin and BSE (curcumin/BSE group). Interleukin-6 (IL-6), tumor necrosis factor-α (TNFα), high-sensitivity c-reactive protein (hs-CRP), total AGE, soluble receptor for AGE (sRAGE), malondialdehyde (MDA), plasma phospholipid fatty acid (PPFA) composition, and non-esterified fatty acids (NEFA) were tested at baseline and after 12 weeks. Results: sRAGE, NEFA, and MDA decreased significantly in both groups, while only the curcumin/BSE group showed a significant decline in total AGE. Only the changes in total AGE and MDA differed significantly between the curcumin/BSE and MD groups. Conclusions. Our data suggest a positive effect of supplementation with curcumin and BSE on glycoxidation and lipid peroxidation in chronically exercising master athletes.

## 1. Introduction

Reactive oxygen species (ROS) have an important role in maintaining homeostasis through immune functions and cellular signals [1], but an excess of ROS has the potential to damage deoxyribonucleic acid (DNA), proteins, and lipids. It has been shown that chronic intensive exercise may be associated with an increased induction of oxidative stress, which in turn has a negative impact on exercise performance and causes muscle damage [2]. Advanced glycation end-products (AGEs) are a heterogeneous group of macromolecules formed by the non-enzymatic glycation of proteins, lipids, and nucleic acids. Excessive glycation in humans can derive from exogenous AGEs ingested with foods, and from endogenous AGEs formed in the body—especially in conditions of chronic hyperglycemia or increased oxidative stress [3]. The harmful effect of AGEs is particularly associated with stiffening of tissues rich in extracellular matrices and long-lived proteins, such as skeletal muscle, tendons, joints, bone, heart, arteries, lung, skin, and lens [4]. Very little is known about how effective dietary intervention and oral antioxidant supplementation may be in reducing oxidative stress in athletes who exercise intensively, but there is some evidence to suggest that the administration of antioxidants and anti-inflammatory nutrients within physiological ranges may reverse exercise-induced oxidative stress and muscle damage [5]. Curcumin, an important constituent of the turmeric (*Curcuma longa*) rhizome, possesses antioxidant and anti-inflammatory activities, and has been used to treat a variety of inflammatory conditions and chronic diseases [6]. It has also been demonstrated that curcumin can reduce the accumulation of AGEs in vitro and in animal models, suggesting that this anti-glycation mechanism may relate to the antioxidant effect of the compound [7]. Extracts of *Boswellia serrata* (BSE) gum resin have also demonstrated anti-inflammatory properties, suppressing local tissue tumor necrosis factor-α (TNFα) and interleukin-1β (IL-1β) in animal models [8].

No studies have ascertained whether oral curcumin supplementation has antioxidant and anti-glycation effects in the medium-term in master athletes chronically exercising intensively, who are particularly exposed to increased glycoxidative processes. Hence, our study aimed to establish whether curcumin and BSE supplementation for 3 months, combined with a Mediterranean diet (MD), could affect plasma levels of markers of oxidative stress, inflammation, and glycation in a cohort of well-trained healthy master cyclists. 

## 2. Patients and Methods

### 2.1. Participants and Study Design

Forty-seven healthy male athletes aged 46 ± 8 years took part in this study after giving their written informed consent. The study complied with the Helsinki Declaration, and the local institutional Ethics Committee approved the study protocol. The trial was designed as a controlled, randomized, parallel group study.

Inclusion criteria were no history of cardiovascular disease or allergies, and no prior use of drugs or food supplements. Exclusion criteria were use of tobacco products, alcohol consumption, recent surgery, diabetes mellitus, hypertension, and chronic inflammatory diseases.

Table 1 shows the anthropometric features of the subjects enrolled in the study, including body composition, assessed using bioelectrical impedance analysis at baseline in all subjects. The participants volunteering for the study were cyclists with an average experience of 8 ± 2 years of competing in the nonprofessional category. They had usually been cycling about 200 km a week and 10,000/12,000 km a year. They were instructed not to change their lifestyle during the trial as regards exercise, diet, and other routine activities, and they were asked not to take any other medicinal herbs or drugs. The athletes were not involved in any special exercise sessions, because the aim of the study was to assess the effect of medium-term curcumin and BSE supplementation in conditions of real-life physical activity.

At the baseline visit, we used 24-h recall—a self-reporting method for collecting data on eating behavior and for measuring energy intake by means of structured interviews—as described elsewhere [9]. An isocaloric Mediterranean-style dietary pattern was recommended to all subjects, with particular recommendation to avoid foods naturally containing curcumin. The athletes were assigned to two groups by means of a simple randomization procedure using a computer-generated random binary list. Group 1 consisted of 22 subjects given a MD alone (MD group); and Group 2 consisted of 25 subjects given a MD plus Fitomuscle^®^ (ForFarma, Rome, Italy), a nutraceutical-combined pill (NCP) containing curcumin and BSE (curcumin/BSE group). One tablet of Fitomuscle^®^ contains 50 mg of turmeric Phytosome^®^—corresponding to 10 mg of curcumin—and 140 mg of *Boswellia* extract, corresponding to 105 mg of boswellic acids. Phytosome^®^ (registered by Indena, Milan, Italy) relies on an advanced formulation technology for delivering substances of herbal origin: this involves the dispersion of the drug in natural phospholipids to improve its absorption after oral administration [10]. The new formulation enables higher systemic levels of curcumin to be obtained than with other formulations [10].

Adherence to the treatment was ascertained by means of pill counts on the product returned at the follow-up visit.

### 2.2. Dietary Intake

Nutrients and food intake were measured using the Willett Food Frequency Questionnaire, which has been validated for a wide range of ages [11]. Full instructions were given on how to complete the questionnaire, together with a list of 120 foods, for each of which there was a full description of the usual serving size. Food preparation was also considered in order to include all the ingredients used. Participants were asked to keep a detailed record of their weekly food consumption, and they completed the questionnaire three times during the study, at the beginning, after the first month, and at the end. They were asked to record the amount of food they consumed and their food preparation methods. Participants were provided with pictures of standard meals and portion sizes to enable them to estimate the size of their portions. All completed questionnaires were checked by a dietitian for accuracy and completeness. The data in the questionnaires were assessed with the aid of a database for us in nutritional analysis (Medimatica S.r.l. 2004, Colonnella, Italy). A trained dietician taught participants how to complete the questionnaires and quantify their food servings. The dietary records were analyzed using the computerized nutritional analysis system Science Fit Diet 200A (Sciencefit, Athens, Greece).

### 2.3. Blood Sampling

The following markers were tested in blood and urine samples obtained at the baseline and after 12 weeks: interleukin-6 (IL-6), tumor necrosis factor-α (TNFα), high-sensitivity c-reactive protein (hs-CRP), total AGE, soluble receptor for AGE (sRAGE), malondialdehyde (MDA), non-esterified fatty acids (NEFA), total cholesterol, low density lipoprotein (LDL) cholesterol, high density lipoprotein (HDL) cholesterol, and triglycerides. Plasma phospholipid fatty acid (PPFA) composition was also ascertained. A competitive enzyme-linked immunosorbent assay (ELISA) was used to assess glycoxidation and inflammatory markers, as described elsewhere [12]. NEFA were assayed using a colorimetric method (Randox^®^, County Antrim, Northern Ireland) with a precision assuring a less than 5% coefficient of variation. PPFA composition was determined after lipid extraction by the method of Folch et al. [13]. All analyses were performed at the laboratory of Padua University Hospital.

### 2.4. Statistical Analysis

Values are expressed in terms of mean ± standard deviation (SD). Findings were considered in terms of the difference at follow-up (12 weeks after starting the study) vis-à-vis each subject’s baseline situation. Negative values thus indicate an actual reduction in a given parameter after the treatment. The statistical significance of any differences induced by the treatment was tested using Student’s *t-*test for paired data. Differences were considered statistically significant when *p* < 0.05 (two-tailed test).

## 3. Results

Data were available for statistical analysis for all 47 subjects randomized. Participants did not differ in age, BMI, or body composition (Table 1). Body composition measurements obtained using bioelectrical impedance did not differ between the baseline and the follow-up 3 months later (Table 1), during which time the two groups of athletes continued to exercise as usual. To assess the effect of curcumin and BSE supplementation, we examined inflammatory markers (IL-6, TNFα, hs-CRP), glycoxidation (AGEs, sRAGE), and lipoxidation (MDA, NEFA). Plasma levels of NEFA and PPFA composition were obtained simultaneously to rule out the possibility of any changes in the two groups’ markers of oxidation and glycation depending on the recommended diet.

As summarized in Table 2, neither group showed any significant changes in inflammatory markers at the follow-up, apart from a slight increase in TNFα in the MD group. sRAGE and NEFA decreased significantly in both groups, while total AGE levels decreased significantly after supplementation with curcumin and BSE, but not in the group given a MD alone. After 3 months, both groups showed a significant decrease in their levels of MDA (chosen as a marker of lipoxidation). Randomization resulted in a significant difference of AGE values at baseline between the two groups; therefore the comparison between the groups was performed by considering differences after minus before treatment (Table 3), obviating the observed differences in AGE: at this analysis, changes in total AGE and MDA differed significantly between the two groups. AGE remained significantly different between the two groups after normalizing for the differences at baseline (−42.41% ± 18.66% in MD + curcumin/BSE group vs. 4.18% ± 22.71% in MD group, *p* < 0.001). No differences emerged between the curcumin/BSE group and MD groups as regards their PPFA composition (Supplementary Materials Table S1).

## 4. Discussion

In our pilot study, medium-term dietary supplementation with curcumin and BSE was effective in reducing markers of glycoxidation and lipoxidation in a group of master athletes chronically exercising intensively. Oral supplementation with curcumin and BSE for 3 months was associated with a significant reduction in total plasma AGEs and MDA.

Studies investigating whether exercise produces clinically significant oxidative stress have reached no definitive conclusions, partly because different types and intensities of exercise were considered, different markers of oxidative stress were measured, and participants had different levels of training [5]. Generally speaking, acute physical activity—be it aerobic or anaerobic—predisposes to an increased ROS production via multiple pathophysiological pathways. It has been suggested that the high ROS levels produced by acute bouts of exercise may be detrimental to the immune system, but recent studies have indicated that chronic exercise—high intensity endurance training in particular—could have a positive impact on redox status, especially because slightly higher levels of ROS may improve anti-oxidant defenses [14]. 

Few studies conducted to date have compared the effects of anaerobic exercise and aerobic exercise on oxidative stress [15,16], however, and only one study compared intermittent endurance and resistance exercise protocols in terms of oxidative stress in middle-aged women [2]. The chronic physical activity of nonprofessional cyclists combines endurance and resistance training sessions, in proportions that it is virtually impossible to quantify precisely in real life, as demonstrated by the lack of literature in this area. 

Curcumin exhibits an antioxidant activity in several in vitro and in vivo models [17]. Curcumin is able to scavenge superoxide anion (∙O_2_^−^∙), hydroxyl radicals (∙OH), H_2_O_2_, singlet oxygen, nitric oxide, peroxynitrite, and peroxyl radicals (ROO∙) [18,19,20,21]. It has also been shown to downregulate the expression of numerous proinflammatory cytokines, including TNFα, Vascular Endothelial Growth Factor (VEGF), and interleukins 1, 2, and 6 [22]. Gum resin extracts of BSE have also revealed anti-inflammatory properties, suppressing local tissue TNFα and IL-1β in animal models [8]. Moreover, an inhibitory activity on AGE has been demonstrated for BSE in vitro and in animal models [23].

Few studies have examined the effect of curcumin supplementation on exercise-induced oxidative stress and exercise performance in animal models [24,25]. One study found curcumin supplementation to be effective in attenuating exercise-induced oxidative stress in young athletes, also improving their antioxidant capacity [26].

AGEs are generated under conditions of hyperglycemia and oxidative stress, or a combination of the two [3]. The engagement of AGEs with their receptor (RAGE) has been implicated in the development of various disorders, including vascular disease [12], and it is related to an amplification of oxidative stress. A number of studies on animals and humans have confirmed an in vivo accumulation of advanced glycation products (AGE) with aging, and this AGE accumulation can occur at the periphery of skeletal muscle fibers, intracellularly, or both [27]. While high levels of circulating AGE seem to predict cardiovascular-related mortality among older communities [28], the precise nature of AGE-induced protein modifications and their clinical significance in the aging process of skeletal muscle remain to be seen. 

No studies published to date have considered the effect of curcumin and BSE on plasma levels of AGEs and sRAGE in humans, and in athletes in particular.

In the present study, master athletes with an average age of 46 years were engaging in non-professional cycling, an activity characterized mainly by high-intensity endurance training associated with short sessions of anaerobic resistance training (i.e., during climbs and shots). We chose these master athletes for our study because they reflect a significant proportion of the physically active population, they were older than competitive athletes, and received less medical and nutritional counseling. Moreover, numerous experimental animal and human data support an AGE accumulation and a depletion of anti-oxidant reserves with aging [29]. To our knowledge, no previous clinical studies have investigated the degree of glycoxidation in this particular population, or the effects of specific nutritional regimens associated with antioxidant supplementation.

The baseline AGEs and sRAGE levels observed in our sample were higher than in a population of healthy individuals of comparable age and ethnicity who were not chronically exercising intensively [30]. In this report of Kerkeni et al., 30 healthy subjects showed average serum AGE of 508.83 ± 119.68 pg/mL and average serum sRAGE of 148.72 ± 32.73 pg/mL. It is particularly difficult to establish standard reference values for serum AGE in healthy subjects, for many reasons: first, the great variability of the plasma concentration ranges between different populations; secondly, the differences between methods used for their blood assay; finally, the influence of the AGE content in foods [31,32]. However, in comparison to population studied by Kerkeni et al., our athletes have about three times higher values of RAGE, and serum AGE values (though determined with a different method) are much higher. This suggests that the type of exercise performed by our athletes can exacerbate chronic levels of oxidative stress, leading to a significant accumulation of AGEs over time. 

In our study, a significant reduction in plasma AGEs was only found in the group of athletes given curcumin/BSE supplementation, while there was a decline in sRAGE in both groups. 

The effect of exercise on sRAGE has been studied a little in healthy subjects and patients with and without diabetes, with contradictory results [33,34]. The physiological function of endogenous sRAGE remains to be seen, since many soluble variants derive from the splicing of the receptor. It is also not clear which of these variants exerts a protective effect by acting as a RAGE scavenger, and which of them amplify the oxidative intracellular signaling of RAGE themselves [35]. In diabetic patients, for example, sRAGE are believed to change dynamically, gradually switching from higher levels in the early stages of inflammation and atherosclerosis to a mild decline in the intermediate stage, before rising again in the acute phase of tissue damage and consequent inflammation [35]. Other authors have hypothesized an increase in the clearance of sRAGE from the circulation as a result of hemodynamic changes induced by a greater physical activity [34], and this latter effect could explain the results of our study. 

By comparison with sRAGE, plasma levels of AGEs are certainly more stable and less affected by acute changes in oxidative stress due to exercise. They therefore better reflect chronic oxidative stress and are more reliable for the purpose of assessing the effects of long-term curcumin supplementation.

The indirect assessment of oxidative stress involves measuring the more stable molecular products formed by the reaction of ROS with certain biomolecules. Some of the common molecular products are the concentrations of oxidation target products, including lipid peroxidation end-products like malondialdehyde (MDA) [36]. A previous study found that intermittent anaerobic exercise, when performed intensively, resulted in acute lipid peroxidation and immune suppression in well-trained women aged 45 to 55 years [2]. 

The Mediterranean diet (MD) has been shown to exert a positive effect on the lipid profile and to have antioxidant properties, especially as regards the dietary intake of high-protein polyunsaturated fatty acids (PUFAs). Malaguti et al. [37] reported that PUFA supplementation might increase susceptibility to lipid peroxidation in volleyball athletes followed up for 2 months: they concluded that the balance between the positive and negative effects of PUFA intake might depend on dosage, and that simply adhering to a MD seems to be a better option. A MD favors a higher intake of PUFAs—particularly of the *n*-3 series—and a lower ratio of *n*-6 to *n*-3 fatty acids than in other diets, thus affecting tissue lipids.

The participants enrolled in this study were all taught to adopt a MD during the 3 months of observation. PPFA composition and serum NEFA adapt dynamically to changes in the dietary fatty acid profile, and we tested these parameters to assess the effect of MD in our athletes. 

As concerns lipid peroxidation, MDA levels dropped in both groups of athletes, but those taking the curcumin/BSE supplement showed a significantly sharper decline than the athletes enrolled in the group only given a MD. After 3 months, serum NEFA levels were similarly reduced in both groups, and we found no significant differences in the qualitative composition of their PUFAs. Therefore, we surmise that MD, although with a positive effect on the drop in NEFA, did not seem to induce any significant increase in PUFAs, which can exclude an eventual contribution to the drop in MDA levels.

Judging from the above observations, it is reasonable to infer that curcumin and BSE supplementation could have a direct and significant effect on plasma lipid peroxidation. Other than that, we found no changes in inflammatory status related to the MD or curcumin/BSE supplementation in either group of athletes, probably because of their already low grade of inflammation.

The main strength of this study lies in its originality. To the best of our knowledge, this is the first study to examine the effect of a food supplement containing curcumin and BSE on plasma levels of sRAGE and AGE in humans. It is also the first study to confirm the positive effect of the supplement in reducing markers of lipid peroxidation in a population of athletes chronically exercising intensively. This has an important implication in nutritional counseling for such athletes, who reflect a considerable proportion of the physically active individuals in the general population and are more prone to higher levels of oxidative stress. The effects of curcumin/BSE supplementation were also assessed over a considerable period of time, while the few studies in the literature considered only a single session of exercise.

Weaknesses of this study include the small number of participants enrolled and our failure to precisely quantify how much the athletes exercised. Having said that, the study was designed to examine people exercising in real life, and to assess the effectiveness of supplementation with curcumin in the medium term.

## 5. Conclusions

Our data suggest a positive effect of curcumin and BSE supplementation for 3 months on glycoxidation and lipid peroxidation in athletes chronically exercising intensively. 

Further studies will test whether treatment with curcumin can result in a reduction of the accumulation of AGEs in muscle tissue, possibly improving muscle performance in the long term.

## Figures and Tables

**Table 1:** Basal characteristics of the patients enrolled.

	Group “MD + Curcumin/BSE”		Group “MD”	
	(*n* = 25)		(*n* = 22)	
	*Pre*	*Post*	*Pre*	*Post*
**Age (year)**	45 ± 9	-	46 ± 8	-
**Weight (kg)**	72.4 ± 8.0	72.4 ± 7.9	71.8 ± 9.6	70.7 ± 21.7
**BMI (kg/m^2^)**	23.7 ± 2.1	23.5 ± 2.0	23.7 ± 2.8	23.0 ± 6.4
**FFM (%)**	81 ± 4	80 ± 4	81 ± 6	79 ± 8
**FM (%)**	19 ± 4	19 ± 4	19 ± 6	21 ± 6

MD: Mediterranean diet; BSE: *Boswellia serrata* extract; BMI: body mass index; FFM: free fat mass; FM: fat mass.

**Table 2:** Parameters of inflammation and glyco-/lipo-oxidative stress evaluated in the two groups, before and after therapeutic intervention.

	MD + Curcumin/BSE		MD	
	*Before*	*After*	*Before*	*After*
**hs-CRP (mg/L)**	2.90 ± 0.2	2.80 ± 0.2	2.60 ± 0.5	2.36 ± 1.0
**NEFA (mmol/L)**	1.05 ± 0.67	0.68 ± 0.52 **	1.24 ± 0.92	0.45 ± 0.18 ***
**sRAGE (pg/mL)**	475.73 ± 141.66	328.50 ± 164.49 ***	430.47 ± 123.59	312.34 ± 156.27 ***
**AGE (µg/mL)**	22.42 ± 18.08	10.83 ± 6.38 ***	9.07 ± 4.22	9.22 ± 4.07
**MDA (µmol/L)**	0.16 ± 0.09	0.05 ± 0.05 ***	0.10 ± 0.03	0.03 ± 0.01 ***
**IL-6 (pg/mL)**	17.62 ± 29.76	17.55 ± 29.58	23.76 ± 53.00	41.88 ± 50.69
**TNFα (pg/mL)**	7.29 ± 5.11	8.28 ± 5.42	7.05 ± 4.61	12.67 ± 11.07 *

NEFA: non-esterified fatty acids; sRAGE: soluble receptor for advanced glycation end products; AGE: advanced glycation end products; MDA: malondialdehyde; IL-6: interleukine-6; TNFα: tumor necrosis factor α; hs-CRP: high-sensitivity c-reactive protein. *** *p* < 0.001; ** *p* < 0.01; * *p* < 0.05. Student’s *t*-test for paired data.

**Table 3:** Pre–post changes in biomarkers of inflammation and glyco-lipo-oxidation compared between the two groups.

	MD + Curcumin/BSE	MD	*p* ^†^
**hs-CRP (mg/L)**	0.10 ± 0.2	0.24 ± 0.54	ns
**NEFA (mmol/L)**	−0.36 ± 0.61	−0.79 ± 0.89	ns
**sRAGE (pg/mL)**	−147.23 ± 109.02	−118.13 ± 117.01	ns
**AGE (µg/mL)**	−11.59 ± 12.49	0.15 ± 2.30	<0.001
**MDA (µmol/L)**	−0.10 ± 0.06	−0.07 ± 0.03	<0.02
**IL−6 (pg/mL)**	−0.07 ± 35.18	18.13 ± 59.73	ns
**TNFα (pg/mL)**	0.99 ± 6.88	5.62 ± 11.14	ns

NEFA: non-esterified fatty acids; sRAGE: soluble receptor for advanced glycation end products; AGE: advanced glycation end products; MDA: malondialdehyde; IL-6: interleukine-6; TNFα: tumor necrosis factor α; hsCRP: c-reactive protein. Variables are presented as the mean (±SD) change from baseline. ^†^ Data were compared using Student’s *t*-test for unpaired data. ns: not statistically significant.

## Supplementary Materials

The following are available online at http://www.mdpi.com/2072-6643/8/11/745/s1. Table S1: Percent differences (after treatment minus before treatment, normalized to before treatment value) in serum fatty acid profile of the two groups of subjects, before and after treatment. Negative values indicate a decrease of the parameter in comparison to before treatment.

## Abbreviations

The following abbreviations are used in this manuscript:

ROS	Reactive oxygen species
AGEs	Advanced glycation end-products
BSE	Boswellia serrate
MD	Mediterranean diet
IL-6	interleukin-6
TNFα	tumor necrosis factor-α
hs-CRP	high-sensitivity c-reactive protein
sRAGE	soluble receptor for AGE
MDA	malondialdehyde
NEFA	non-esterified fatty acids
PPFA	plasma phospholipid fatty acid
NCP	nutraceutical-combined pill
PUFA	polyunsaturated fatty acid
FFM	fat-free mass
FM	fat mass
BMI	body mass index
