# Supplementary material for: Curcumin and Boswellia serrata Modulate the Glyco-Oxidative Status and Lipo-Oxidation in Master Athletes
Source: Nutrients. 2016 Nov 21;8(11):745. doi: 10.3390/nu8110745 (PMC5133128; doi:10.3390/nu8110745)
Supplement: Supplementary file 1 [file nutrients-08-00745-s001.docx]

Supplementary Materials: Curcumin and
*Boswellia serrata* Modulate the Glyco-Oxidative Status and Lipo-Oxidation in Master Athletes

Nino Cristiano Chilelli, Eugenio Ragazzi, Romina Valentini, Chiara Cosma, Stefania Ferraresso, Annunziata Lapolla and Giovanni Sartore

**Table S1.** Percent differences (after treatment minus before treatment, normalized to before treatment value) in serum fatty acid profile of the two groups of subjects, before and after treatment. Negative values indicate a decrease of the parameter in comparison to before treatment.

| **Fatty Acid** | **MD + Curcumin/BSE** | **MD** | ***p* ^†^** |
| --- | --- | --- | --- |
| **14:00** | 21.27 ± 164.63 | 1.80 ± 32.21 | 0.5885 |
| **16:00** | 1.21 ± 5.97 | 2.79 ± 7.49 | 0.4248 |
| **16:01 *n*7** | −21.90 ± 27.16 | −6.48 ± 35.69 | 0.1001 |
| **18:00** | 2.50 ± 7.40 | 2.20 ± 14.65 | 0.9283 |
| **18:1 *n*9** | −6.51 ± 10.67 | −7.11 ± 9.86 | 0.8439 |
| **18:2 *n*6** | 1.60 ± 7.04 | −1.51 ± 24.00 | 0.5387 |
| **18:3 n3** | 8.36 ± 63.74 | 7.03 ± 39.52 | 0.9331 |
| **20:1 *n*9** | 3.05 ± 17.14 | 43.73 ± 165.36 | 0.2271 |
| **20:3 *n*6** | 5.46 ± 20.06 | 10.68 ± 26.61 | 0.4485 |
| **20:4 *n*6** | 2.15 ± 11.88 | 3.26 ± 17.58 | 0.7978 |
| **20:5 *n*3** | 8.17 ± 61.28 | 30.90 ± 17.58 | 0.2463 |
| **22:4 *n*6** | −1.44 ± 15.90 | 1.94 ± 25.08 | 0.5785 |
| **22:5 *n*3** | 0.84 ± 25.00 | 7.38 ± 25.62 | 0.3808 |
| **24:00:00** | 30.46 ± 51.06 | 23.25 ± 32.80 | 0.5737 |
| **22:6 *n*3** | 6.04 ± 23.84 | 0.55 ± 25.07 | 0.4462 |
| **24:01:00** | 56.04 ± 78.14 | 72.70 ± 67.88 | 0.4424 |

Variables are presented as the mean ± SD percent change from baseline. ^†^ Student’s *t*-test for unpaired data, comparison between the percent differences in the two treatment groups. BSE: *Boswellia serrata* extract; MD: Mediterranean diet.
